# Hypofractionated image-guided breath-hold SABR (Stereotactic Ablative Body Radiotherapy) of liver metastases – clinical results

**DOI:** 10.1186/1748-717X-7-92

**Published:** 2012-06-18

**Authors:** Judit Boda-Heggemann, Dietmar Dinter, Christel Weiss, Anian Frauenfeld, Kerstin Siebenlist, Ulrike Attenberger, Martine Ottstadt, Frank Schneider, Ralf-Dieter Hofheinz, Frederik Wenz, Frank Lohr

**Affiliations:** 1 Department of Radiation Oncology, University Medical Center Mannheim, University of Heidelberg, Mannheim, Germany; 2 Institute of Diagnostic Radiology and Nuclear Medicine, University Medical Center Mannheim, University of Heidelberg, Mannheim, Germany; 3 Department of Biomathematics and Medical Statistics, University Medical Center Mannheim, University of Heidelberg, Mannheim, Germany; 4 III. Department of Internal Medicine, University Medical Center Mannheim, University of Heidelberg, Mannheim, Germany; 5 Department of Radiation Oncology, University Medical Center Mannheim, University of Heidelberg, Theodor-Kutzer-Ufer 1-3, 68167, Mannheim, Germany

**Keywords:** Hypofractionated image-guided breath-hold SABR, Liver metastases, Local control, Survival, Toxicity

## Abstract

**Purpose:**

Stereotactic Ablative Body Radiotherapy (SABR) is a non-invasive therapy option for inoperable liver oligometastases. Outcome and toxicity were retrospectively evaluated in a single-institution patient cohort who had undergone ultrasound-guided breath-hold SABR.

**Patients and methods:**

19 patients with liver metastases of various primary tumors consecutively treated with SABR (image-guidance with stereotactic ultrasound in combination with computer-controlled breath-hold) were analysed regarding overall-survival (OS), progression-free-survival (PFS), progression pattern, local control (LC), acute and late toxicity.

**Results:**

PTV (planning target volume)-size was 108 ± 109cm^3^ (median 67.4 cm^3^). BED2 (Biologically effective dose in 2 Gy fraction) was 83.3 ± 26.2 Gy (median 78 Gy). Median follow-up and median OS were 12 months. Actuarial 2-year-OS-rate was 31%. Median PFS was 4 months, actuarial 1-year-PFS-rate was 20%. Site of first progression was predominantly distant. Regression of irradiated lesions was observed in 84% (median time to detection of regression was 2 months). Actuarial 6-month-LC-rate was 92%, 1- and 2-years-LC-rate 57%, respectively. BED2 influenced LC. When a cut-off of BED2 = 78 Gy was used, the higher BED2 values resulted in improved local control with a statistical trend to significance (p = 0.0999). Larger PTV-sizes, inversely correlated with applied dose, resulted in lower local control, also with a trend to significance (p-value = 0.08) when a volume cut-off of 67 cm^3^ was used.

No local relapse was observed at PTV-sizes < 67 cm^3^ and BED2 > 78 Gy. No acute clinical toxicity > °2 was observed. Late toxicity was also ≤ °2 with the exception of one gastrointestinal bleeding-episode 1 year post-SABR. A statistically significant elevation in the acute phase was observed for alkaline-phosphatase; in the chronic phase for alkaline-phosphatase, bilirubine, cholinesterase and C-reactive protein.

**Conclusions:**

A trend to statistically significant correlation of local progression was observed for BED2 and PTV-size. Dose-levels BED2 > 78 Gy cannot be reached in large lesions constituting a significant fraction of this series. Image-guided SABR (igSABR) is therefore an effective non-invasive treatment modality with low toxicity in patients with small inoperable liver metastases.

## Background/Introduction

Standard therapy for solitary liver metastases is surgical resection [1]. For medically or technically inoperable patients, therapy options include radiofrequency-, microwave- and cryo-/laser-ablation, alcohol-injection, transarterial chemoembolisation and stereotactic ablative radiotherapy (SABR) [2,3]. SABR is the only truly non-invasive approach, combining high efficacy with minimal side effects in the oligometastatic situation [4].

Dose-escalation has improved local control [5]. High-dose liver-SABR is a technical challenge because of respiration-induced movement and vicinity of radiosensitive Organs-At-Risk (OAR [6]). The main problems are precise tumor immobilisation as well as fast and reliable visualisation and localisation of the lesions. Irradiation during free-breathing with the Internal Target Volume concept and abdominal compression would require a relatively larger PTV compared to breath-hold defined PTV (Planning Target Volume, due to breathing motion [7]).

Breath-hold techniques enable tumor immobilisation and the delivery of high doses to the PTV while maximally sparing the OARs [8]. Computer-controlled breath-hold with the ABC®-system (Active Breathing Coordinator, Elekta, Crawley, U.K.) in combination with daily image-guidance has been shown to be feasible for liver targets [9]. Upper abdominal tumors can be rapidly visualised and localised with stereotactic ultrasound (B-mode acquisition and targeting, BAT®, Nomos) [10-12], either directly or relying on surrogate structures (liver capsule, vessels), or with on-board cone-beam-CT (CBCT) [13] with a repeat breath-hold approach [14].

Data of large patient cohorts on local control (LC) and survival after liver SABR are relatively rare due to lack of large randomised studies. Given the novelty of SABR, studies (both prospective and retrospective) with long-term follow-up data are still rare.

In this retrospective evaluation, we intended to assess outcome and toxicity in a unique single-institution series of patients who received volume image-guided breath-hold liver SABR.

Overall-survival (OS), progression-free-survival (PFS), local control (LC), acute and late toxicity based on clinical and laboratory parameters and CTC/LENT-SOMA (Common Toxicity Criteria/ Late Effects on Normal Tissues; Subjective Objective Management Analysis) scales were evaluated.

## Patients and methods

### Patients

Between 2005–2010, 22 lesions in 19 consecutive patients (15 male, 4 female; median age 69ys, range 39-87ys) were irradiated (one re-irradiation, in 2 patients 2 lesions in 1 PTV) after informed consent. All lesions were liver metastases of various primary tumors (10 colorectal, 1 prostate, 2 breast, 2 melanoma, 1 GIST (Gastrointestinal Stromal Tumor), 1 oesophagus, 1 hepatocellular carcinoma, 1 NSCLC (Non-Small-Cell Lung Cancer)) which were considered to be technically or medically inoperable by an interdisciplinary tumor-conference. The cohort can be characterised by a relatively large number of patients with poor prognosis (5 patients with extrahepatic metastases and 2 patients with uncontrolled primaries).

### Radiotherapy planning and treatment

Planning CT scans were acquired with a spiral-CT (Somatom Emotion, thereafter Somatom Volume Zoom; Siemens, Erlangen, Germany) with i.v. contrast, in the portal-venous phase after an initial patient training session in inspiratory breath-hold at approximately 70% of vital capacity with ABC® [12]. Radiotherapy planning was initially performed as 3-dimensional conformal RadioTherapy (3D-CRT) with OTP, Theranostic GmbH, Solingen, Germany and thereafter with Intensity Modulated RadioTherapy (IMRT) with Monaco®, Elekta. A pre-therapeutic MRI or PET-CT was performed and the information was used during planning for GTV (Gross Tumor Volume) definition. PTV was calculated from GTV by adding a 5 mm margin radially and 10 mm in the craniocaudal direction to compensate residual intrafractional error of the ABC®-based positioning [15].

Organ contours and surrogate structures needed for ultrasound-based image guidance (portal vein, liver veins, and liver capsule) were identified by a physician [10,12] and were exported to BAT®

Dose prescription was initially performed to the isocenter (3D-CRT, typically in the vicinity of the median dose) and later as the median dose in the PTV (IMRT) with the 90% isodose line covering the PTV. Dose constraints for OAR were as follows: mean dose to each kidney was restricted to 10 Gy, maximal dose in the spinal cord to 25 Gy. Hot spots in >0.5 cm^3^ of hollow organs (stomach/small intestine/oesophagus) were restricted to a maximum of 8 Gy/fraction for treatments with ≤3 fractions and a maximum of 6 Gy/fraction for treatments with 5 fractions. 50% of healthy liver tissue was kept below 10 Gy [16]. Implementing results from published literature reports regarding dose escalation and fractionation, dose to the patients was adjusted during the reported period and varied between single-fraction doses of 24-30 Gy initially (depending on tumor and liver volume) and various hypofractionated regimens with the current, final protocol prescribing 5x12Gy every other day.

Biologically effective dose in 2 Gy fractions (BED2) was calculated using the linear-quadratic model with an assumed α/β ratio of 10 as described [6]:

(1) $$\text{BED}2\;\text{= Dx}\left(\text{d}+\alpha /\beta \right)/\left(2+\alpha /\beta \right)\text{.}$$

**Figure 1:**
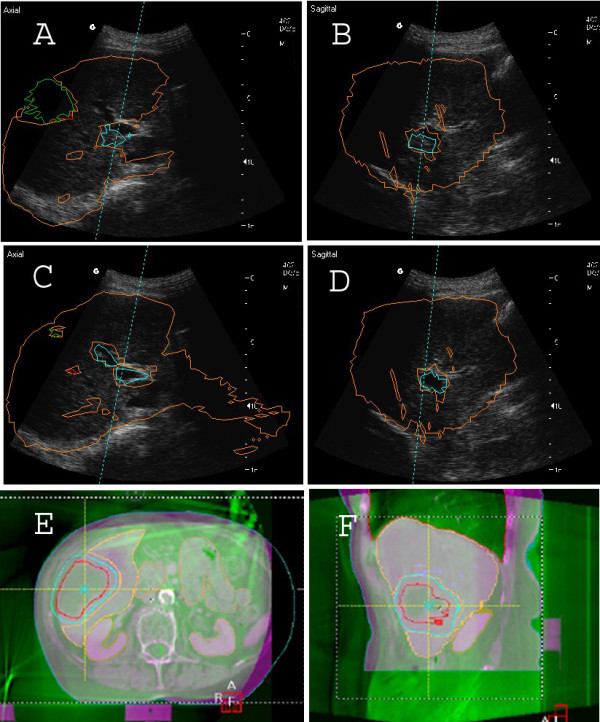
**Daily positioning with BAT®.** Before (**A-B**) and after (**C-D**) position correction. Surrogate structures and PTV are marked with colors: orange, liver; green, PTV; blue, portal vein; red, liver veins. **E-F**: Alternative repeat breath-hold positioning with CBCT. Green, CBCT; magenta, planning-CT.

Patients were treated on a linac with 6MV photons (Synergy®, Elekta). Daily image-guidance was performed with BAT® and beginning in 2005 with additional CBCT with image acquisition in repeated breath-hold (XVI®, X-Ray Volume Imaging, Elekta; [17]). CT-structures, contours of PTV and OAR/surrogate structures were imported into the BAT®- and XVI®-system as 3D structures in DICOM (Digital Imaging and Communication in Medicine) format. Although the lesion to be treated could not always directly be visualized by ultrasound, the liver as a whole as well as surrogate structures (portal vein, liver veins) could be precisely visualized and matched and served as surrogates for positioning of the target [10,12] (Figure 1A-D). No implanted fiducials were used for EPID localisation to establish a completely non-invasive treatment modality [10].

Linac, BAT® and ABC® were not connected by interfaces. Breath-hold and imaging or beam were synchronized manually. When CBCT was used (used for initial positioning/assessment of overall geometric situation and rarely exclusively if BAT® was not available, Figure 1E-F), planning-CT images were matched online with the daily CBCT images acquired in repeat breath-hold [17] using manual fusion and with respect to soft-tissue anatomy. On-line surveillance of breath-hold was performed based on the continuous acquisition of MV-frames (EPID; Electronic Portal Imaging Device) during irradiation allowing position verification of the diaphragm. Intrafractional error was controlled/corrected with BAT® at least two times per fraction.

Patient follow-up (FU) was scheduled 6 weeks after radiotherapy and every 3 months thereafter with clinical examination, liver-MRI with i.v. contrast (or CT/PET-CT, if MRI contraindicated) and laboratory values. An assessment of tumor response was performed using the RECIST (Response Evaluation and Criteria in Solid Tumors) criteria. Response was graded as complete response (CR), partial response (PR), stable disease (SD) or progression.

Acute toxicity was defined as symptoms observed in the first 90 days after radiotherapy, with later symptoms being scored as late toxicity.

### Data analysis

OS (Overall-Survival), PFS (Progression-Free-Survival) and LC (Local Control) were recorded and subject to actuarial analysis. Acute (first 90 days) and late toxicity (>90 days) was evaluated based on clinical symptoms and laboratory values (graded based on the CTC-scale in the acute phase and on the LENT-SOMA criteria in the late phase.

Recorded clinical endpoints/symptoms were general condition, abdominal pain, appetite, nausea, vomiting, fever and skin symptoms for assessing acute toxicity; abdominal pain, hepatomegaly, edema, weight gain due to edema/ascites, vigilance, bleedings, ascites and rib fracture for late toxicity.

The following laboratory values were analysed: ALAT (alanine-aminotransferase), ASAT (aspartate-aminotransferase), GGT (gamma-glutamyl-transferase), LDH (lactate-dehydrogenase), AP (alkaline-phosphatase), BR (bilirubine), aPTT (activated partial thromboplastine-time), thrombocyte-count, serum albumin, CHE (cholinesterase), prothrombin activity, blood urea, CRP (C-reactive-protein).

PTV-coverage was analysed based on relevant parameters (D99 (dose encompassing 99% of the PTV), minimal, maximal, mean and median PTV-dose. Volume of healthy liver tissue was documented. Depth of breath-hold and breath-hold duration was also evaluated.

### Statistics

Statistical analysis was performed with the SAS-software, release 9.01 (SAS, Cary, NC, USA). OS was calculated from the day of irradiation until either the day of death (event) or the day of the last FU (follow-up, censored data). PFS was calculated from the day of irradiation until either the day of relapse or death (events) or the last FU without relapse (censored data when at the last FU the patient lived without any evidence of progression). LC was calculated from the day of irradiation until either the day of local progression or death (event) or the last FU without local progression (censored data when at the last FU the patient lived without any evidence of local progression). Kaplan-Meier-curves for OS, PFS and LC were calculated in order to assess median survival/control times. Correlation of local progression as a binary parameter and PTV size and BED2 was analysed with the logistic regression test. Correlation of the local control time with PTV size and BED2 was analysed by the Kaplan-Meier log-rank test. Differences between pre- and post-therapeutic laboratory values were tested using the Dunnett-test. Test results were considered significant when p-values were <0.05.

## Results

### Radiotherapy data

All patients managed to achieve sufficient repeat breath-hold with ABC® for an IMRT (3 patients) or 3DCRT (16 patients) radiotherapy session. Breath-hold time was 17.2 ± 3.6 seconds. Depth of breath-hold was 1.5 ± 0.4 litres (threshold value in ABC®). Volume of healthy liver tissue was 1401 ± 350 cm^3^.

BED2 was 83.3 ± 26.2 Gy (median 78 Gy, range 44-150 Gy). 7 patients were treated with a BED2 of <78 Gy, 12 patients with a BED2 of ≥78 Gy. PTV dose-data for different fractionation schedules are summarised in Table 1. PTV-size was 108 ± 109cm^3^ (median 67.4 cm^3^, range 11–419 cm^3^).

**Table 1:** Mean, median, minimal and maximal absolute doses to the PTV for different fractionation regimens

**PTV dose parameter**	**Dose (Gy)**			
	**(Mean value ± Standard deviation)**			
	***single-fraction***	***2 fractions***	***3 fractions***	***5 fractions***
	***SABR* (n = 12)**	**(n = 2)**	**(n = 3)**	**(n = 5)**
**D**_**mean**_	26,07 ± 1,81	27,16 ± 3,87	33,92 ± 1,37	59,51 ± 0,58
**D**_**median**_	26,11 ± 1,79	27,12 ± 3,88	34,34 ± 1,47	59,89 ± 0,66
**D**_**min**_	23,9 ± 1,85	21,23 ± 3,31	26,56 ± 2,46	50,92 ± 8,00
**D**_**max**_	27,26 ± 2,06	29,3 ± 5,44	36,3 ± 0,89	62,48 ± 1,24
**D**_**99**_	24,2 ± 2,62	25,65 ± 4,0	32,6 ± 2,55	57,54 ± 3,19

D_99_: Dose encompassing 99% of the PTV.

Abbreviations: D_min_, minimal dose; D_max_, maximal dose; D_median_, median dose; D_mean_, mean dose, PTV, Planning Target Volume.

### Follow-up (FU), Overall-Survival (OS), Progression-free-survival (PFS) and Local Control (LC)

Median FU was 12 months (14 ± 12 months; MV ± SD, mean value ± standard deviation). Median FU was 12 months (18 ± 15 months) for living patients. 5 patients are alive, 13 patients have died and one patient has been lost to FU (released from jail, not traceable).

**Figure 2:**
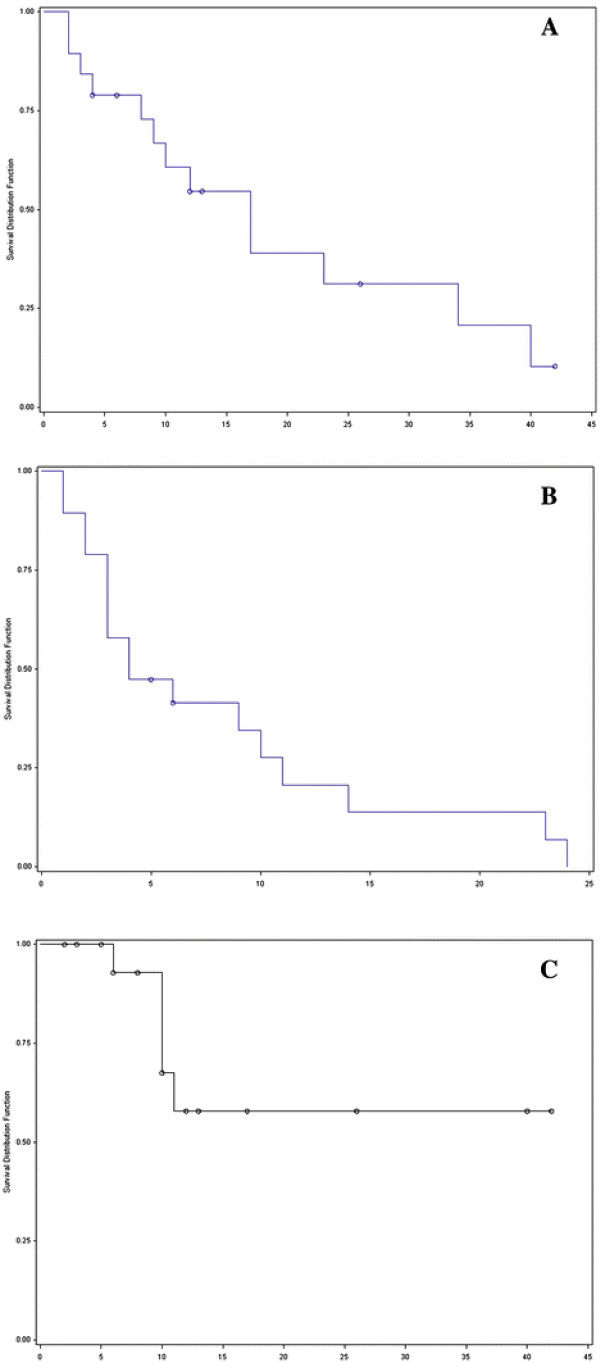
**Kaplan-Meier-curves for all patients.A.**) Overall-survival. **B**.) Progression-free-survival. **C**.) Local control.

Actuarial median OS was 12 months (15 ± 13 months; Figure 2A). Actuarial 2-year-OS was 31%. Cause of death was systemic metastases outside the liver in 5 patients, progression of the primary tumor (oesophagus/colorectal) in 5 patients, an episode of acute gastrointestinal bleeding in one patient and hepatic insufficiency in one patient, mainly induced by hemochromatosis and liver cirrhosis that had been prevalent over several years. In one patient, the cause of death could not be determined unequivocally but brain metastases were suspected clinically when therapeutic measures were halted because of deteriorating general condition.

Median PFS was 4 months (8 ± 8 months; Figure 2B), actuarial 1-year PFS was 20%. Progression pattern was mainly distant (a total 17 patients experienced systemic progression; 10 patients had a second liver metastasis, 9 patients had extrahepatic distant metastases in brain/lung/bones/kidney/adrenal-gland/spleen/peritoneum/abdominal-wall/abdominal-lymph nodes).

Median LC has not yet been reached. Actuarial 6-month LC-rate was 92%, 2-year LC rate was 57% (Figure 2C). Most patients died due to systemic metastases or progress of the primary (77%) with locally controlled irradiated lesions.

**Figure 3:**
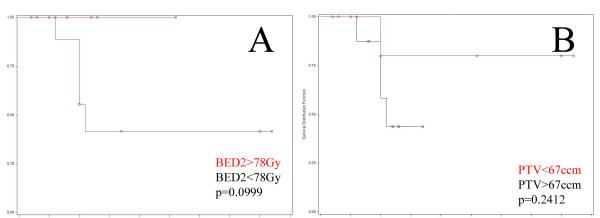
**A) Local control of patients with BED2 ≥ 78 Gy (red) and <78 Gy (black), p = 0.0999 (trend to significance; Kaplan-Meier log-rank test).B**) Local control of patients with PTV < 67 cm^3^ (red) and ≥67 cm^3^ (black), p = 0.2412, not significant.

Correlation of Local Progression as a binary parameter and PTV size and BED2 was analysed with the logistic regression test. PTV size has shown a trend towards lower local control in larger lesions (p = 0.0894). However, correlation of local control time to PTV size (cut-off 67ccm) with the Kaplan Meier Log-rank test has not shown statistical significance (p = 0.2412; Figure 3A).

Local recurrences were only observed for patients receiving a BED2 ≤ 78 Gy (5 patients). BED2 did not show a significant correlation in the logistic regression (p = 0.1926) but a trend to significance at a dose cut-off of 78 Gy with the Kaplan-Meier log-rank test (p = 0.0999; Figure 3B).

In 16 patients, size-reduction of the irradiated lesion was observed, in 3 patients, lesions remained unchanged. Median time to response was 2 months (3 ± 1 months, typically at first reported FU). Complete response of the irradiated lesion was observed in 5 cases. Later local progression after initial regression was observed in 5 patients after a median of 10 months.

**Figure 4:**
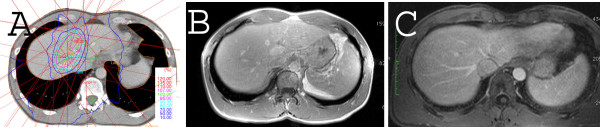
**Radiation plan, pretreatment- and posttreatment MRI of a patient with two small liver metastases of a melanoma treated with 1x26Gy in 1 PTV.** The lesions remain locally controlled (follow-up of 4 years).

A representative patient example is shown in Figure 4.

### Acute toxicity

No acute toxicity > CTC grade 2 was observed. The following symptoms were detected: slight upper-abdominal pain (3patients), appetite loss (4patients), nausea (3patients), slight vomiting (2patients) and fatigue (4patients). Fever was observed in only 1 patient, however, this was considered a dexamethasone side-effect.

One patient developed a clinically indolent but radiologically evident and lab-proven cholangitis, which was successfully treated with interventional endoscopy. No clinical signs of pneumonitis and no acute gastrointestinal bleedings were observed.

A significant elevation compared to pre-therapeutic values in the acute phase was observed only for AP (p = .0274), indicating an intrahepatic cholestasis, which, however, in most patients was clinically unapparent (in one patient radiologically detectable). ALAT, ASAT, GGT, LDH, BR, aPTT, thrombocyte count, albumin, CHE, prothrombin activity, blood urea and CRP did not show significant changes.

### Chronic toxicity

Late toxicity was difficult to analyse in this cohort of patients with advanced tumor stages due to progressive disease causing symptoms similar to radiation-associated processes/radiation sequelae. Several patients who developed multiple liver metastases, suffered from ascites, edema, weight gain and other signs of hepatic insufficiency. Patients without hepatic progression did not develop any chronic toxicity regarding the upper abdomen. No rib fractures were observed.

One patient experienced an episode of endoscopically confirmed gastrointestinal bleeding 1 year post-igSABR. An anatomical correlation of the endoscopic report about the bleeding with the radiation plan was performed. No spatial correlation could be found, however, a “matching” of an endoscopy report with planning CT is certainly not possible.

A significant elevation compared to pre-therapeutic values in the chronic phase was observed for AP (p = 0.0016), BR (p = 0.0222), CHE (p = 0.0056) and CRP (p = 0.0320). A trend to significance was observed for GGT (p = 0.0673). ALAT, ASAT, LDH, aPTT, thrombocytes, albumine, prothrombin activity and blood urea did not show significant changes.

## Discussion

In SABR of liver metastases, dose escalation has been shown to be essential to increase local control [1]. Technical improvement of planning and delivery have enabled dose escalation, within the confines of liver tolerance dose-volume constraints [18]. A relatively high interfractional accuracy was achievable with frame-based positioning in free-breathing with abdominal compression. Daily soft-tissue image-guided frameless stereotaxy with either ultrasound or cone-beam CT have mainly replaced the frame-based setup [19]. Daily sonographic imaging of the target lesion or intrahepatic surrogate structures allows fast imaging in breath-hold and in the future even tracking [10,12,14]. There are no large prospective studies evaluating the clinical results of SABR of the liver. The present cohort is the first clinically reported series of igSABR using an ultrasound-guided computer-controlled breath-hold setup.

Radiation-induced change in the liver include Radiation-Induced Liver Disease (RILD) with a syndrome of anicteric ascites and hepatomegaly, elevation of transaminases and reactivation of virus hepatitis [20]. Despite the detected histological and radiological radiation reactions, clinical symptoms after liver SABR have been reported in several studies to be low with maximum side effect reaching grade 3 [3]. Similar to literature data, clinical acute and late toxicity did not exceed CTC/ LENT-SOMA grade 2 in our collective. Solely one episode of gastric bleeding 1-year post-SBRT was detected. Based on localisation of the treated lesion and the source of bleeding, association with igSABR is unlikely but cannot be excluded with certainty in retrospect.

Outcome cannot be compared according to primary malignancy in the published series because of small numbers of patients treated for individual disease. Varying fractionation schedules in different series and also within one series, however, can be compared calculating BED2. In addition, most studies evaluate mixed cohorts of lung and liver SABR without a clear differentiation of these two populations.

Lesions receiving BED2 < 78 Gy did not achieve satisfactory local control in this series. We analysed the available studies in the literature also with calculating BED2 and compared these to our data (Table 2, [5,21-26]). Comparing all published studies, a maximum 1-yr local control rates of 57% was reported, if BED2 < 50 Gy was applied in large PTVs (range up to >400ccm).

**Table 2:** Comparison of available studies in the literature

**Study**	**Irradiated liver metastases (n)**	**Nominal total dose/single dose (Gy)**	**BED2 (Gy)**	**Lesion size (ccm)**	**Median FU (months)**	**1-year LC rate (%)**
Katz et al., 2007 [21]	69	30-55/2-6	40-73*	0.1-950*	14.5	76
McCammon et al., 2009 [5]	81	3 dose levels:	3 levels*:	2.8-370	8.2	
		1.) < 36/12	1.) 39			1.) 40
		2.) 36-54/12-18	2.) 39-58			2.) 88
		3.) 54/18	3.) 58			3.) 100
Milano et al., 2008 [22]	121	10-20/10-20	16-50	0.2-422	Not stated	57
Goodmann et al., 2010 [23]	40	18-30/18-30	42-100*	0.8-146	17	77
Iwata et al., 2010 [24]	12	50-55/5-5.5	62-71*	0.9-22*	14.5	86
Rusthoven et al., 2009 [25]	63	36-60/12-20	66-150*	<113*	16	95
Lee et al., 2009 [26]	68	42-60/7-10	59-100*	1.19-3000	10.8	71
Current series JBH et al.	22	24-60/24-12	44-150	11-419	12	57

BED2 and PTV volumes were calculated by JBH et al. (V = 4/3πr^3^; median/range) in the cases marked with *.

Our cohort with mixed primary tumor entities represented an unfavourable patient subset (several patients had extensive metastatic disease). Fractionation schedule and nominal doses varied over the evaluated period, reflecting escalation of published doses. Doses used initially can be regarded as too low in retrospect with the current knowledge. The daily repositioning technique used for this series was unique (computer-controlled breath hold in combination with sonographic imaging) compared to similar analyses in the literature. Median survival of 12 months and a 1-year LC rate of 57% are, however, comparable to literature data. Due to the relatively small and heterogeneous collective, we could not establish a formal dose-effect relationship. No local relapse was observed at PTV-sizes smaller as 67 cm^3^ and doses >78 Gy BED_2_ and a trend to statistically significant correlation was observed for both parameters. This dose level could not be reached due to threshold doses in large lesions constituting a significant fraction of this series. That the results are not highly statistically significant, is probably due to the small patient number and relatively large range of applied BED2 (between 44-150 Gy) and PTV-sizes (between 21-419 cm^3^).

Steep dose gradients and precise intrafractional repositioning before each fraction is possible with the currently available commercial methods. Non-invasive markerless intrafractional monitoring of tumor position is, however, not yet possible with commercially available systems with ultrasound-based online monitoring being developed [27]. Precise positioning and online surveillance will probably allow further reduction of safety margins (for an average CTV size of 113ccm the reduction of safety margin of 5 mm would spare 66ccm healthy liver tissue). This would enable irradiation of larger lesions with higher doses.

Results may further be improved by parallel administration of chemotherapeutic agents for radiosensitization. Large studies are needed to optimize clinical outcome and characterise radiological and clinical side effects.

These data show that, if the application technique, lesion size and dose to OAR allow the application of doses approaching the ablation threshold dose calculated by Fowler and Tome [28], then SABR of the liver with photons is an effective, non-invasive and safe treatment method. Similar results have been observed for SABR of the lung [29,30]. For large lesions (>70 cm^3^), if no BED2 > 80 Gy can be reached without a high risk of side effects, results are still not satisfactory. Limiting factor for dose escalation in liver SABR is the radiation tolerance of healthy liver tissue. After introduction of modern highly precise techniques with steep dose gradients (VMAT (Volumetric Modulated Arc Therapy) [31,32], IMRT) with daily 3D image guidance, maximal efficacy of photon-based SBRT has been reached.

New radiotherapy techniques may provide further optimization of dose distributions [33]. Detection of intrafraction motion by online tracking may facilitate applying sufficiently high doses to larger lesions.

## Conclusions

Breath-hold image guided SABR of liver metastases is an effective and safe treatment modality for inoperable liver metastases.

No local relapse was observed at PTV-sizes < 67 cm^3^ and BED2 > 78 Gy and a trend to statistically significant correlation with local control was observed for both parameters. However, this dose level cannot be reached in large lesions constituting a significant fraction of this series.

## Competing interest

This work was in part supported by a grant from Elekta Inc. and Nomos.

There are no conflicts of interest.

## Authors’ contributions

JBH, AF: data acquisition. JBH: manuscript drafting. DD, UA: interpretation of radiological data (MRI, CT). CW: statistical evaluation. KS, FL: radiation treatment planning. FS: radiation treatment planning, QA. RH: chemotherapy administration, treatment follow-up. MO: interpretation of laboratory data. FW, FL, JBH and all others: final approval of MS text. All authors read and approved the final manuscript.
